# 2-[*N*-(4-{4-[(*E*)-(2-Hydroxybenzyl­idene)amino]phenoxy}phenyl)carbox­imidoyl]phenol

**DOI:** 10.1107/S1600536811007847

**Published:** 2011-03-05

**Authors:** Gholam Hossein Shahverdizadeh, Edward R. T. Tiekink

**Affiliations:** a Department of Chemistry, Faculty of Science, Islamic Azad University, Tabriz Branch, Tabriz, PO Box 1655, Iran; b Department of Chemistry, University of Malaya, 50603 Kuala Lumpur, Malaysia

## Abstract

The mol­ecular structure of the title Schiff base compound, C_26_H_20_N_2_O_3_, shows that respective methyl­idene residues are almost coplanar with the adjacent terminal benzene ring owing to the presence of intra­molecular O—H⋯N hydrogen bonds [the N—C—C—C torsion angles are −6.6 (7) and −6.7 (7)°]. However, twists are exhibited about each methyl­idene and respective benzene ring connected to the central O atom; the dihedral angles formed between the two inner and two outer benzene rings are 54.6 (2) and 45.6 (3)°, respectively. The conformation about each of the C=N bonds [1.285 (5) and 1.295 (5) Å] is *E*. In the crystal, extensive C—H⋯π contacts involving all benzene rings results in the formation of layers in the *ac* plane.

## Related literature

For related structures of Schiff base ligands, see: Chu & Huang (2007 ▶); Xu *et al.* (2008 ▶); Prasath *et al.* (2010 ▶). For details of crystallization from a U-tube under non-ambient conditions, see: Harrowfield *et al.* (1996 ▶).
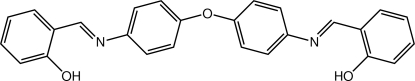


## Experimental

### 

#### Crystal data

C_26_H_20_N_2_O_3_
*M* *_r_* = 408.44
Monoclinic, 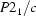

*a* = 6.0234 (9) Å
*b* = 46.225 (7) Å
*c* = 9.4371 (11) Å
β = 129.636 (7)°
*V* = 2023.5 (5) Å^3^
*Z* = 4
Mo *K*α radiation
μ = 0.09 mm^−1^
*T* = 293 K
0.35 × 0.24 × 0.08 mm

#### Data collection

Bruker APEXII CCD diffractometer
Absorption correction: multi-scan (*SADABS*; Sheldrick, 1996 ▶) *T*_min_ = 0.856, *T*_max_ = 1.000
10647 measured reflections
3567 independent reflections
1468 reflections with *I* > 2σ(*I*)
*R*_int_ = 0.089

#### Refinement

*R*[*F* ^2^ > 2σ(*F* ^2^)] = 0.069
*wR*(*F* ^2^) = 0.242
*S* = 1.05
3567 reflections
283 parameters
H-atom parameters constrained
Δρ_max_ = 0.33 e Å^−3^
Δρ_min_ = −0.31 e Å^−3^

### 

Data collection: *APEX2* (Bruker, 2007 ▶); cell refinement: *SAINT* (Bruker, 2007 ▶); data reduction: *SAINT*; program(s) used to solve structure: *SHELXS97* (Sheldrick, 2008 ▶); program(s) used to refine structure: *SHELXL97* (Sheldrick, 2008 ▶); molecular graphics: *ORTEP-3* (Farrugia, 1997 ▶) and *DIAMOND* (Brandenburg, 2006 ▶); software used to prepare material for publication: *publCIF* (Westrip, 2010 ▶).

## Supplementary Material

Crystal structure: contains datablocks global, I. DOI: 10.1107/S1600536811007847/hg5006sup1.cif

Structure factors: contains datablocks I. DOI: 10.1107/S1600536811007847/hg5006Isup2.hkl

Additional supplementary materials:  crystallographic information; 3D view; checkCIF report

## Figures and Tables

**Table 1:** Hydrogen-bond geometry (Å, °) *Cg*1–*Cg*4 are the centroids of the C1—C6, C8–C13, C14–C19 and C21–C26 rings, respectively.

*D*—H⋯*A*	*D*—H	H⋯*A*	*D*⋯*A*	*D*—H⋯*A*
O1—H1*o*⋯N1	0.82	1.88	2.602 (5)	147
O3—H3*o*⋯N2	0.82	1.86	2.589 (5)	147
C23—H23⋯*Cg*1^i^	0.93	2.90	3.556 (6)	129
C26—H26⋯*Cg*1^ii^	0.93	2.90	3.566 (6)	129
C16—H16⋯*Cg*2^iii^	0.93	2.76	3.469 (6)	133
C19—H19⋯*Cg*2^iv^	0.93	2.86	3.582 (5)	135
C9—H9⋯*Cg*3^i^	0.93	2.76	3.462 (5)	133
C12—H12⋯*Cg*3^ii^	0.93	2.88	3.584 (5)	134
C3—H3⋯*Cg*4^iv^	0.93	2.87	3.512 (6)	128
C6—H6⋯*Cg*4^iii^	0.93	2.95	3.626 (7)	131

Symmetry codes: (i) 
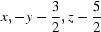
; (ii) 
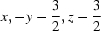
; (iii) 
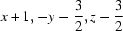
; (iv) 
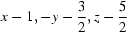
.

**Fig. 1.**
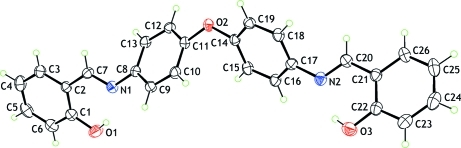
The molecular structure of (I) showing the atom-labelling scheme and displacement ellipsoids at the 35% probability level.

**Fig. 2.**
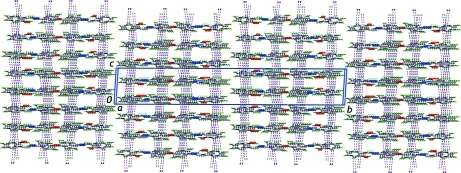
View approximately in projection down the a axis of the unit-cell contents for (I). The C—H···π contacts are shown as purple dashed lines.

## supplementary crystallographic information

### Comment

In continuation of structural studies of Schiff base ligands (Prasath *et al.*, 2010), attention is now directed to evaluating the structures of flexible derivatives (Chu & Huang, 2007; Xu *et al.*, 2008) which have been shown, for example, to lead to helical coordination polymers (Chu & Huang, 2007). In this context the title compound (I) was prepared and characterized crystallographically.

The molecule of (I) is twisted at both the central O atom, and about each of the methylidene residues. Thus, the dihedral angle formed between the least-squares planes through the benzene rings directly connected to the central O atom is 54.6 (2) °. Similarly, the dihedral angles formed between the terminal benzene ring and the adjacent benzene ring are 51.6 (2) ° for each of C1–C6/C8–C13 and C14–C19/C21–C26. Finally, the dihedral angle formed between the terminal benzene rings is 45.6 (3) °. The conformation about each of the C═N bonds [N1═C7 is 1.285 (5) Å and N2═C20 is 1.295 (5) Å] is *E*. The presence of intramolecular O—H···N hydrogen bonds, Table 1, ensures co-planarity between the respective terminal benzene rings and methylidene residues as reflected in the C1—C2—C7—N1 and N2—C20—C21—C22 torsion angles of -6.6 (7) and -6.7 (7) °. respectively.

The crystal packing is dominated by C—H···π interactions, Table 1, whereby each of the four benzene rings accepts two such contacts. The result is the formation of layers in the *ac* plane, Fig. 2.

### Experimental

A solution of 4,4'-diaminodiphenyl ether (10 mmol) in ethanol (50 ml) was added drop wise to a solution of salicylaldehyde (20 mmol) in ethanol (50 ml). The mixture was stirred for 6 h. The resulting solution was filtered to obtain a Schiff base which was dried. Crystals of the title compound were obtained by using the branched tube method (Harrowfield *et al.*, 1996) where the Schiff base (5 mmol) was placed in the arm to be heated. Methanol was carefully added to fill both arms, and then the arm to be heated was placed in a bath at 333 K. After 2 days, yellow crystals were deposited in the cooler arm, which were filtered, washed with water and air dried.

### Refinement

The O– and C-bound H atoms were geometrically placed (O—H = 0.82 Å and C–H = 0.93 Å) and refined as riding with *U_iso_*(H) = *yU_eq_*(parent atom) for *y* = 1.5 (O) and 1.2 (C).

### Crystal data

**Table:**

C_26_H_20_N_2_O_3_	*F*(000) = 856
*M**_r_* = 408.44	*D*_x_ = 1.341 Mg m^−^^3^
Monoclinic, *P*2_1_/*c*	Mo *K*α radiation, λ = 0.71073 Å
Hall symbol: -P 2ybc	Cell parameters from 806 reflections
*a* = 6.0234 (9) Å	θ = 2.6–20.9°
*b* = 46.225 (7) Å	µ = 0.09 mm^−^^1^
*c* = 9.4371 (11) Å	*T* = 293 K
β = 129.636 (7)°	Prism, yellow
*V* = 2023.5 (5) Å^3^	0.35 × 0.24 × 0.08 mm
*Z* = 4	

### Data collection

**Table:**

Bruker APEXII CCD diffractometer	3567 independent reflections
Radiation source: fine-focus sealed tube	1468 reflections with *I* > 2σ(*I*)
graphite	*R*_int_ = 0.089
φ and ω scans	θ_max_ = 25.0°, θ_min_ = 1.8°
Absorption correction: multi-scan (*SADABS*; Sheldrick, 1996)	*h* = −7→7
*T*_min_ = 0.856, *T*_max_ = 1.000	*k* = −54→31
10647 measured reflections	*l* = −11→11

### Refinement

**Table:**

Refinement on *F*^2^	Secondary atom site location: difference Fourier map
Least-squares matrix: full	Hydrogen site location: inferred from neighbouring sites
*R*[*F*^2^ > 2σ(*F*^2^)] = 0.069	H-atom parameters constrained
*wR*(*F*^2^) = 0.242	*w* = 1/[σ^2^(*F*_o_^2^) + (0.0859*P*)^2^] where *P* = (*F*_o_^2^ + 2*F*_c_^2^)/3
*S* = 1.05	(Δ/σ)_max_ < 0.001
3567 reflections	Δρ_max_ = 0.33 e Å^−^^3^
283 parameters	Δρ_min_ = −0.31 e Å^−^^3^
0 restraints	Extinction correction: *SHELXL*, Fc^*^=kFc[1+0.001xFc^2^λ^3^/sin(2θ)]^-1/4^
Primary atom site location: structure-invariant direct methods	Extinction coefficient: 0.012 (2)

### Special details

**Table:**

Geometry. All s.u.'s (except the s.u. in the dihedral angle between two l.s. planes) are estimated using the full covariance matrix. The cell s.u.'s are taken into account individually in the estimation of s.u.'s in distances, angles and torsion angles; correlations between s.u.'s in cell parameters are only used when they are defined by crystal symmetry. An approximate (isotropic) treatment of cell s.u.'s is used for estimating s.u.'s involving l.s. planes.
Refinement. Refinement of *F*^2^ against ALL reflections. The weighted *R*-factor *wR* and goodness of fit *S* are based on *F*^2^, conventional *R*-factors *R* are based on *F*, with *F* set to zero for negative *F*^2^. The threshold expression of *F*^2^ > 2σ(*F*^2^) is used only for calculating *R*-factors(gt) *etc*. and is not relevant to the choice of reflections for refinement. *R*-factors based on *F*^2^ are statistically about twice as large as those based on *F*, and *R*- factors based on ALL data will be even larger.

### Fractional atomic coordinates and isotropic or equivalent isotropic displacement parameters (Å^2^)

**Table:**

	*x*	*y*	*z*	*U*_iso_*/*U*_eq_	
O1	0.9581 (7)	0.39134 (8)	0.7320 (5)	0.0577 (11)	
H1o	0.8694	0.3762	0.7075	0.087*	
O2	0.1380 (6)	0.24995 (7)	0.6475 (4)	0.0383 (9)	
O3	0.7882 (8)	0.10965 (8)	0.5591 (5)	0.0560 (11)	
H3o	0.7480	0.1245	0.5856	0.084*	
N1	0.5304 (8)	0.35847 (8)	0.6345 (5)	0.0365 (10)	
N2	0.5548 (8)	0.14148 (9)	0.6585 (5)	0.0384 (10)	
C1	0.7827 (10)	0.41395 (11)	0.6882 (6)	0.0405 (13)	
C2	0.4989 (10)	0.40991 (10)	0.6172 (6)	0.0339 (11)	
C3	0.3264 (11)	0.43421 (11)	0.5699 (6)	0.0428 (13)	
H3	0.1367	0.4318	0.5221	0.051*	
C4	0.4323 (12)	0.46186 (11)	0.5928 (7)	0.0525 (15)	
H4	0.3142	0.4779	0.5581	0.063*	
C5	0.7160 (13)	0.46527 (12)	0.6678 (7)	0.0545 (15)	
H5	0.7903	0.4838	0.6866	0.065*	
C6	0.8909 (12)	0.44174 (12)	0.7155 (7)	0.0509 (15)	
H6	1.0818	0.4444	0.7661	0.061*	
C7	0.3857 (10)	0.38160 (10)	0.6009 (6)	0.0372 (12)	
H7	0.2021	0.3800	0.5649	0.045*	
C8	0.4255 (9)	0.33115 (10)	0.6359 (6)	0.0315 (11)	
C9	0.4603 (9)	0.30768 (10)	0.5600 (6)	0.0339 (11)	
H9	0.5465	0.3104	0.5071	0.041*	
C10	0.3696 (10)	0.28052 (10)	0.5618 (6)	0.0366 (12)	
H10	0.3878	0.2651	0.5067	0.044*	
C11	0.2507 (9)	0.27635 (9)	0.6465 (6)	0.0322 (12)	
C12	0.2179 (9)	0.29916 (10)	0.7254 (6)	0.0338 (12)	
H12	0.1379	0.2962	0.7820	0.041*	
C13	0.3047 (9)	0.32640 (10)	0.7197 (6)	0.0349 (12)	
H13	0.2823	0.3419	0.7726	0.042*	
C14	0.2517 (10)	0.22375 (10)	0.6487 (6)	0.0313 (12)	
C15	0.5385 (10)	0.21951 (10)	0.7314 (6)	0.0377 (13)	
H15	0.6668	0.2350	0.7849	0.045*	
C16	0.6329 (10)	0.19221 (10)	0.7340 (6)	0.0352 (12)	
H16	0.8251	0.1895	0.7878	0.042*	
C17	0.4477 (9)	0.16882 (10)	0.6583 (6)	0.0339 (12)	
C18	0.1595 (9)	0.17341 (10)	0.5739 (6)	0.0357 (12)	
H18	0.0316	0.1579	0.5211	0.043*	
C19	0.0608 (9)	0.20082 (10)	0.5677 (6)	0.0349 (12)	
H19	−0.1331	0.2038	0.5092	0.042*	
C20	0.4768 (10)	0.11806 (10)	0.6915 (6)	0.0389 (13)	
H20	0.3650	0.1194	0.7276	0.047*	
C21	0.5609 (9)	0.08991 (10)	0.6732 (6)	0.0348 (12)	
C22	0.7033 (10)	0.08644 (11)	0.6017 (6)	0.0386 (12)	
C23	0.7609 (11)	0.05915 (12)	0.5731 (7)	0.0485 (14)	
H23	0.8522	0.0570	0.5231	0.058*	
C24	0.6833 (11)	0.03516 (12)	0.6185 (7)	0.0555 (16)	
H24	0.7213	0.0168	0.5983	0.067*	
C25	0.5489 (12)	0.03808 (12)	0.6941 (7)	0.0549 (15)	
H25	0.5031	0.0218	0.7284	0.066*	
C26	0.4831 (11)	0.06532 (11)	0.7186 (6)	0.0443 (13)	
H26	0.3868	0.0673	0.7652	0.053*	

### Atomic displacement parameters (Å^2^)

**Table:**

	*U*^11^	*U*^22^	*U*^33^	*U*^12^	*U*^13^	*U*^23^
O1	0.046 (2)	0.053 (3)	0.078 (3)	0.0038 (19)	0.041 (2)	−0.001 (2)
O2	0.0476 (19)	0.0225 (19)	0.051 (2)	0.0009 (16)	0.0340 (18)	0.0014 (14)
O3	0.072 (3)	0.046 (3)	0.075 (3)	−0.003 (2)	0.059 (2)	−0.002 (2)
N1	0.043 (2)	0.031 (2)	0.036 (2)	−0.003 (2)	0.026 (2)	−0.0042 (18)
N2	0.036 (2)	0.034 (3)	0.038 (2)	−0.0007 (19)	0.020 (2)	−0.0003 (18)
C1	0.046 (3)	0.038 (3)	0.040 (3)	−0.002 (3)	0.029 (3)	−0.003 (2)
C2	0.036 (3)	0.029 (3)	0.036 (3)	−0.002 (2)	0.023 (2)	−0.001 (2)
C3	0.047 (3)	0.034 (3)	0.046 (3)	0.000 (3)	0.029 (3)	−0.001 (2)
C4	0.070 (4)	0.030 (3)	0.053 (4)	0.003 (3)	0.037 (3)	−0.002 (3)
C5	0.071 (4)	0.039 (4)	0.053 (4)	−0.021 (3)	0.039 (3)	−0.007 (3)
C6	0.054 (3)	0.051 (4)	0.053 (4)	−0.008 (3)	0.036 (3)	−0.001 (3)
C7	0.035 (3)	0.035 (3)	0.042 (3)	0.002 (2)	0.025 (2)	−0.002 (2)
C8	0.032 (3)	0.028 (3)	0.031 (3)	0.001 (2)	0.019 (2)	0.000 (2)
C9	0.040 (3)	0.033 (3)	0.036 (3)	0.000 (2)	0.027 (2)	−0.002 (2)
C10	0.048 (3)	0.029 (3)	0.039 (3)	0.006 (2)	0.031 (3)	0.000 (2)
C11	0.036 (3)	0.028 (3)	0.028 (3)	0.002 (2)	0.019 (2)	0.001 (2)
C12	0.037 (3)	0.034 (3)	0.034 (3)	0.003 (2)	0.025 (2)	−0.002 (2)
C13	0.044 (3)	0.028 (3)	0.036 (3)	0.002 (2)	0.027 (3)	−0.006 (2)
C14	0.038 (3)	0.027 (3)	0.032 (3)	0.002 (2)	0.024 (3)	0.001 (2)
C15	0.040 (3)	0.032 (3)	0.033 (3)	−0.005 (2)	0.019 (3)	−0.001 (2)
C16	0.032 (3)	0.029 (3)	0.037 (3)	−0.002 (2)	0.019 (2)	−0.002 (2)
C17	0.036 (3)	0.037 (3)	0.030 (3)	0.001 (2)	0.022 (2)	−0.001 (2)
C18	0.039 (3)	0.026 (3)	0.039 (3)	−0.006 (2)	0.023 (2)	−0.005 (2)
C19	0.032 (3)	0.036 (3)	0.034 (3)	−0.003 (2)	0.020 (2)	0.000 (2)
C20	0.044 (3)	0.036 (3)	0.039 (3)	−0.003 (3)	0.028 (3)	−0.001 (2)
C21	0.033 (3)	0.031 (3)	0.037 (3)	−0.001 (2)	0.020 (2)	−0.001 (2)
C22	0.039 (3)	0.032 (3)	0.041 (3)	0.002 (2)	0.024 (3)	0.000 (2)
C23	0.045 (3)	0.046 (4)	0.052 (4)	0.006 (3)	0.030 (3)	−0.003 (3)
C24	0.049 (3)	0.037 (4)	0.059 (4)	0.006 (3)	0.025 (3)	−0.005 (3)
C25	0.061 (4)	0.032 (3)	0.056 (4)	−0.002 (3)	0.030 (3)	0.008 (3)
C26	0.052 (3)	0.033 (3)	0.048 (3)	−0.003 (3)	0.032 (3)	0.005 (2)

### Geometric parameters (Å, °)

**Table:**

O1—C1	1.350 (5)	C10—H10	0.9300
O1—H1o	0.8200	C11—C12	1.376 (6)
O2—C14	1.388 (5)	C12—C13	1.378 (6)
O2—C11	1.399 (5)	C12—H12	0.9300
O3—C22	1.356 (5)	C13—H13	0.9300
O3—H3o	0.8200	C14—C19	1.381 (6)
N1—C7	1.285 (5)	C14—C15	1.382 (6)
N1—C8	1.416 (6)	C15—C16	1.378 (6)
N2—C20	1.295 (5)	C15—H15	0.9300
N2—C17	1.418 (6)	C16—C17	1.381 (6)
C1—C6	1.388 (7)	C16—H16	0.9300
C1—C2	1.395 (6)	C17—C18	1.389 (6)
C2—C3	1.396 (6)	C18—C19	1.385 (6)
C2—C7	1.438 (6)	C18—H18	0.9300
C3—C4	1.383 (6)	C19—H19	0.9300
C3—H3	0.9300	C20—C21	1.447 (6)
C4—C5	1.379 (7)	C20—H20	0.9300
C4—H4	0.9300	C21—C26	1.397 (6)
C5—C6	1.375 (7)	C21—C22	1.398 (7)
C5—H5	0.9300	C22—C23	1.380 (6)
C6—H6	0.9300	C23—C24	1.374 (7)
C7—H7	0.9300	C23—H23	0.9300
C8—C9	1.387 (6)	C24—C25	1.386 (8)
C8—C13	1.392 (6)	C24—H24	0.9300
C9—C10	1.374 (6)	C25—C26	1.383 (7)
C9—H9	0.9300	C25—H25	0.9300
C10—C11	1.385 (6)	C26—H26	0.9300
			
C1—O1—H1o	109.5	C12—C13—H13	119.5
C14—O2—C11	121.5 (4)	C8—C13—H13	119.5
C22—O3—H3o	109.5	C19—C14—C15	120.2 (4)
C7—N1—C8	120.5 (4)	C19—C14—O2	115.6 (4)
C20—N2—C17	120.7 (4)	C15—C14—O2	124.1 (4)
O1—C1—C6	118.5 (5)	C16—C15—C14	119.5 (5)
O1—C1—C2	121.6 (4)	C16—C15—H15	120.2
C6—C1—C2	120.0 (5)	C14—C15—H15	120.2
C1—C2—C3	118.7 (5)	C15—C16—C17	121.3 (4)
C1—C2—C7	121.7 (4)	C15—C16—H16	119.3
C3—C2—C7	119.5 (4)	C17—C16—H16	119.3
C4—C3—C2	121.3 (5)	C16—C17—C18	118.6 (5)
C4—C3—H3	119.4	C16—C17—N2	118.7 (4)
C2—C3—H3	119.4	C18—C17—N2	122.5 (4)
C5—C4—C3	118.9 (5)	C19—C18—C17	120.6 (4)
C5—C4—H4	120.6	C19—C18—H18	119.7
C3—C4—H4	120.6	C17—C18—H18	119.7
C6—C5—C4	121.1 (5)	C14—C19—C18	119.7 (4)
C6—C5—H5	119.4	C14—C19—H19	120.2
C4—C5—H5	119.4	C18—C19—H19	120.2
C5—C6—C1	120.0 (5)	N2—C20—C21	121.0 (5)
C5—C6—H6	120.0	N2—C20—H20	119.5
C1—C6—H6	120.0	C21—C20—H20	119.5
N1—C7—C2	122.0 (4)	C26—C21—C22	118.9 (5)
N1—C7—H7	119.0	C26—C21—C20	118.9 (5)
C2—C7—H7	119.0	C22—C21—C20	122.0 (4)
C9—C8—C13	118.4 (4)	O3—C22—C23	118.4 (5)
C9—C8—N1	118.5 (4)	O3—C22—C21	121.1 (4)
C13—C8—N1	122.9 (4)	C23—C22—C21	120.5 (5)
C10—C9—C8	121.1 (4)	C24—C23—C22	119.9 (5)
C10—C9—H9	119.5	C24—C23—H23	120.0
C8—C9—H9	119.5	C22—C23—H23	120.0
C9—C10—C11	119.4 (4)	C23—C24—C25	120.6 (5)
C9—C10—H10	120.3	C23—C24—H24	119.7
C11—C10—H10	120.3	C25—C24—H24	119.7
C12—C11—C10	120.7 (4)	C26—C25—C24	119.9 (5)
C12—C11—O2	115.3 (4)	C26—C25—H25	120.1
C10—C11—O2	123.8 (4)	C24—C25—H25	120.1
C11—C12—C13	119.4 (4)	C25—C26—C21	120.1 (5)
C11—C12—H12	120.3	C25—C26—H26	119.9
C13—C12—H12	120.3	C21—C26—H26	119.9
C12—C13—C8	121.0 (4)		
			
O1—C1—C2—C3	−178.2 (4)	C11—O2—C14—C19	−151.5 (4)
C6—C1—C2—C3	1.9 (7)	C11—O2—C14—C15	31.5 (6)
O1—C1—C2—C7	5.1 (7)	C19—C14—C15—C16	−0.7 (7)
C6—C1—C2—C7	−174.8 (5)	O2—C14—C15—C16	176.3 (4)
C1—C2—C3—C4	−0.2 (7)	C14—C15—C16—C17	−1.1 (7)
C7—C2—C3—C4	176.6 (4)	C15—C16—C17—C18	1.8 (7)
C2—C3—C4—C5	−1.5 (8)	C15—C16—C17—N2	177.5 (4)
C3—C4—C5—C6	1.6 (8)	C20—N2—C17—C16	140.5 (4)
C4—C5—C6—C1	0.1 (8)	C20—N2—C17—C18	−44.0 (6)
O1—C1—C6—C5	178.3 (5)	C16—C17—C18—C19	−0.7 (7)
C2—C1—C6—C5	−1.8 (7)	N2—C17—C18—C19	−176.2 (4)
C8—N1—C7—C2	173.4 (4)	C15—C14—C19—C18	1.7 (7)
C1—C2—C7—N1	−6.6 (7)	O2—C14—C19—C18	−175.5 (4)
C3—C2—C7—N1	176.7 (4)	C17—C18—C19—C14	−1.0 (7)
C7—N1—C8—C9	140.4 (4)	C17—N2—C20—C21	173.5 (4)
C7—N1—C8—C13	−43.6 (6)	N2—C20—C21—C26	177.0 (4)
C13—C8—C9—C10	2.1 (6)	N2—C20—C21—C22	−6.7 (7)
N1—C8—C9—C10	178.3 (4)	C26—C21—C22—O3	−178.7 (4)
C8—C9—C10—C11	−2.3 (7)	C20—C21—C22—O3	5.0 (7)
C9—C10—C11—C12	1.2 (7)	C26—C21—C22—C23	1.3 (7)
C9—C10—C11—O2	176.5 (4)	C20—C21—C22—C23	−175.1 (4)
C14—O2—C11—C12	−152.1 (4)	O3—C22—C23—C24	178.6 (4)
C14—O2—C11—C10	32.4 (6)	C21—C22—C23—C24	−1.3 (8)
C10—C11—C12—C13	0.0 (7)	C22—C23—C24—C25	−0.4 (8)
O2—C11—C12—C13	−175.7 (4)	C23—C24—C25—C26	2.2 (8)
C11—C12—C13—C8	−0.2 (7)	C24—C25—C26—C21	−2.3 (8)
C9—C8—C13—C12	−0.9 (6)	C22—C21—C26—C25	0.6 (7)
N1—C8—C13—C12	−176.9 (4)	C20—C21—C26—C25	177.0 (4)

### Hydrogen-bond geometry (Å, °)

**Table:**

Cg1–Cg4 are the centroids of the C1—C6, C8–C13, C14–C19 and C21–C26 rings, respectively.

**Table:**

*D*—H···*A*	*D*—H	H···*A*	*D*···*A*	*D*—H···*A*
O1—H1o···N1	0.82	1.88	2.602 (5)	147
O3—H3o···N2	0.82	1.86	2.589 (5)	147
C23—H23···Cg1^i^	0.93	2.90	3.556 (6)	129
C26—H26···Cg1^ii^	0.93	2.90	3.566 (6)	129
C16—H16···Cg2^iii^	0.93	2.76	3.469 (6)	133
C19—H19···Cg2^iv^	0.93	2.86	3.582 (5)	135
C9—H9···Cg3^i^	0.93	2.76	3.462 (5)	133
C12—H12···Cg3^ii^	0.93	2.88	3.584 (5)	134
C3—H3···Cg4^iv^	0.93	2.87	3.512 (6)	128
C6—H6···Cg4^iii^	0.93	2.95	3.626 (7)	131

Symmetry codes: (i) *x*, −*y*−3/2, *z*−5/2; (ii) *x*, −*y*−3/2, *z*−3/2; (iii) *x*+1, −*y*−3/2, *z*−3/2; (iv) *x*−1, −*y*−3/2, *z*−5/2.
